# A practical approach to common hand problems

**DOI:** 10.4102/phcfm.v2i1.162

**Published:** 2010-07-27

**Authors:** Ian Couper

**Affiliations:** 1 Faculty of Health Science, University of the Witwatersrand, South Africa

*The hand book: A practical approach to common hand problems* was edited jointly by internationally known hand surgeon, Ulrich Mennen and occupational therapist, Corrianne van Velze. Both have worked in the field of hand surgery for many years, published extensively and presented numerous papers at conferences.

The book is written well and thoughtfully, combining the experiences of both surgeon and therapist, which makes it unique. The original text, which was a compilation of the work of many authors, has been revised extensively by Mennen and van Velze. The result is a comprehensive book, which should be of great benefit to both the family physician and the hand therapist. The hand book will also be useful as a prescribed book for both occupational and physiotherapists, medical students, nurses, registrars in orthopaedic and plastic surgery and family medicine registrars doing advanced-skills training. The hand book will certainly be a useful resource for rural hospitals.

The book begins with basic structural anatomy, followed by the clinical evaluation of the hand, wrist, elbow and shoulder. The chapters cover a wide range of topics that include the injured hand, fractures and joints, flexor and extensor tendon surgery, peripheral nerve injuries, tendon transfers, hand infections, skin and scarring, tumours, arthritis and congenital anomalies. Each chapter has an introduction followed by a detailed description of the condition and clinical management from both an orthopaedic and hand therapy point of view and comprehensively explains surgical procedures and follow-up hand therapy. References, further recommended reading and questions are provided at the end of each chapter.

One problem is the use of terminology related to disability. This is important as it can reflect one’s attitude and understanding of disability. The correct terms were clearly outlined in the WHO *International classification of functioning, disability and health* in 2001. In *The hand book*, the word ‘handicap’ has been used incorrectly, instead of impairment; handicap should refer to the obstacles that society places on individuals with a health condition or impairment, for example, a building with only stairs at the entrance prohibiting people in wheelchairs to enter.

*The hand book* is highly recommended as a useful and practical guide to the management of a range of hand injuries and conditions.

**FIGURE 1:**
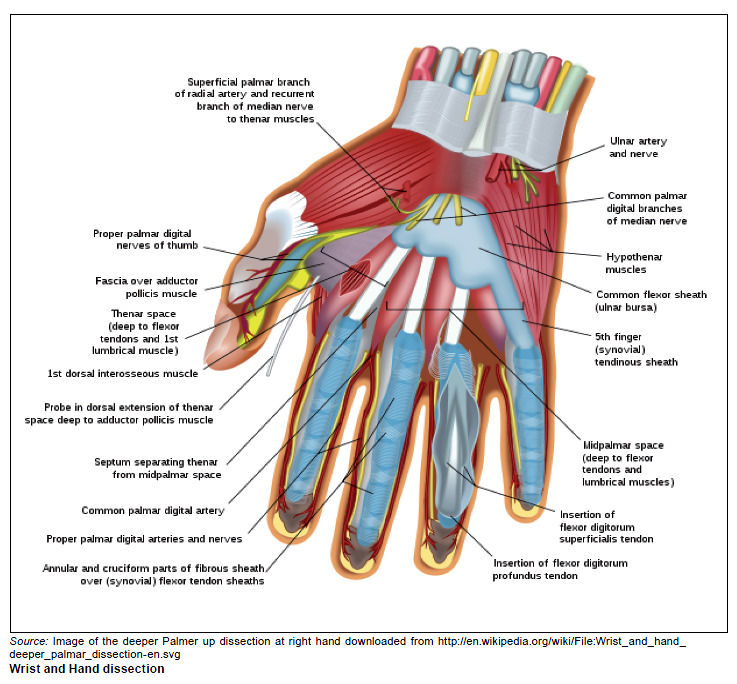
Wrist and Hand dissection. *Source:* Image of the deeper Palmer up dissection at right hand downloaded from http://en.wikipedia.org/wiki/File:Wrist_and_hand_ deeper_palmar_dissection-en.svg

